# The Antiviral Activities and Mechanisms of Marine Polysaccharides: An Overview

**DOI:** 10.3390/md10122795

**Published:** 2012-12-12

**Authors:** Wei Wang, Shi-Xin Wang, Hua-Shi Guan

**Affiliations:** 1 Key Laboratory of Marine Drugs, Ministry of Education, Ocean University of China, Qingdao 266003, China; E-Mails: shixinwang87@gmail.com (S.-X.W.); hsguan@ouc.edu.cn (H.-S.G.); 2 Shandong Provincial Key Laboratory of Glycoscience & Glycoengineering, Ocean University of China, Qingdao 266003, China

**Keywords:** marine polysaccharides, antiviral activities, mechanisms of action, heparinoidspolysaccharide, structure–activity relationship

## Abstract

Recently, the studies on the antiviral activities of marine natural products, especially marine polysaccharides, are attracting more and more attention all over the world. Marine-derived polysaccharides and their lower molecular weight oligosaccharide derivatives have been shown to possess a variety of antiviral activities. This paper will review the recent progress in research on the antiviral activities and the mechanisms of these polysaccharides obtained from marine organisms. In particular, it will provide an update on the antiviral actions of the sulfated polysaccharides derived from marine algae including carrageenans, alginates, and fucans, relating to their structure features and the structure–activity relationships. In addition, the recent findings on the different mechanisms of antiviral actions of marine polysaccharides and their potential for therapeutic application will also be summarized in detail.

## 1. Introduction

In recent years, the constant outbreak of some emerging or reemerging viral diseases has caused serious harm to human health. During the last decade, the number of antivirals approved for clinical use has been increased from five to more than 30 drugs [1]. Despite these successes, drug efficacy, toxicity, and cost remained unresolved issues, which is particularly large in developing countries due to the relative unavailability of drugs and the continuous emergence of drug resistance. Hence, the development of novel antiviral agents that can be used alone or in combination with existing antivirals is of high importance.

Marine polysaccharides are very important biological macromolecules which widely exist in marine organisms. Marine polysaccharides present an enormous variety of structures and are still under-exploited, thus they should be considered as a novel source of natural compounds for drug discovery [2]. Marine polysaccharides can be divided into different types such as marine animal polysaccharides, plant polysaccharides and microbial polysaccharides according to their different sources. Marine derived polysaccharides have been shown to have a variety of bioactivities such as antitumor, antiviral, anticoagulant, antioxidant, immuno-inflammatory effects and other medicinal properties. In particular, the studies on the antiviral actions of marine polysaccharides and their oligosaccharide derivatives are attracting increasing interests, and marine polysaccharides are paving the way for a new trend in antiviral drugs.

This review presents an overview of recent progress in research on the antiviral activities of marine polysaccharides, relating to their structure features and structure–activity relationships. Moreover, this review will mainly focus on the heparinoid polysaccharides and the sulfated polysaccharides present in seaweed. Recent developments in the mechanisms of antiviral actions of marine polysaccharides and their oligosaccharide derivatives will also be discussed in detail.

## 2. The Classification and Main Structure Features of Marine Polysaccharides

Marine polysaccharides usually exhibit structural features such as sulfate and uronic acid groups, which distinguish them from polysaccharides of terrestrial plants, but are similar to mammalian glycosaminoglycans, such as heparin and chondroitin sulfate [3]. Marine polysaccharides can be classified into three main types: marine animal polysaccharides, marine plant polysaccharides, and marine microbial polysaccharides according to their different sources, and each have different structure features. 

### 2.1. The Main Structure Features of Marine Animal Polysaccharides

Marine animals are rich in polysaccharides, and the polysaccharides derived from marine fishes, shellfishes, and mollusks often possess a wide range of pharmacological activities [4]. The marine animal polysaccharides usually include chitosans derived from crustaceans, chondroitin sulfates from cartilaginous fishes, sulfated polysaccharides from sponge, and glycosaminoglycans from scallops and abalone [5,6].

Chitin, a long-chain polymer of *N*-acetylglucosamine, is one of the most abundant polysaccharides and usually prepared from the shells of crabs and shrimps [7,8]. Chitosan, a partially deacetylated polymer of *N*-acetylglucosamine, is produced commercially by deacetylation of chitin [9]. The molecular weight of commercially produced chitosan is usually between 3800 and 20,000 Daltons. Chitosan is a linear randomly distributed, hetero polysaccharide consisting of β-(1→4)-linked 2-acetamido-2-deoxy-D-glucopyranose and 2-amino-2-deoxy-D-glucopyranose units [2]. Chemical modification of chitin and chitosan can generate new biofunctional products which possess good biological activities and physicochemical properties [10,11,12,13,14]. Moreover, it was reported that the marine polysaccharides isolated from cartilaginous fishes also contain trace neutral mannose, xylose and rhamnose besides the galactosamine and glucuronic acid, which have certain structure-specific properties [15].

Furthermore, Cimino *et al.* [16] reported that rosacelose, a new anti-HIV polysaccharide composed of glucose and fucose sulfate, could be isolated from the aqueous extract of the marine sponge *Mixylla rosacea*. They also found that this marine polysaccharide has a linear polysaccharide structure mainly composed of 4,6-disulfated 3-*O*-glycosylated α-D-glucopyranosyl and 2,4-disulfated 3-*O*-glycosylated α-L-fucopyranosyl residues (in a 3:1 molar ratio) [16]. Moreover, it was reported that one kind of sugar polymer which contains hexosamine, hexuronic acid, and fucose sulfate could be separated from *Apostichopus japonicus selenka* [17]. In a word, marine animal polysaccharides have extremely broad distribution, and exist in almost all marine animal tissues and organs. 

### 2.2. The Main Structure Features of Marine Plant Polysaccharides

The marine plant polysaccharides especially the seaweed polysaccharides are widely distributed in the ocean, occurring from the tide level to considerable depths, free-floating or anchored, which are the most abundant polysaccharides in marine organisms. Moreover, the polysaccharide content of seaweed is very high, accounting for more than 50% of the dry weight, thus the seaweed polysaccharides are very important resources for the development of marine polysaccharide drugs. The principal cell wall polysaccharides in green seaweeds are ulvans, those in red seaweeds are agarans and carrageenans, and those in brown seaweeds are alginates and fucans, as well as the storage polysaccharide laminarin [18,19].

Alginates are the major constituent of brown seaweeds’ cell walls and are linear acidic polysaccharides composed with a central backbone of poly-D-glucuronic acid (G blocks), poly-D-mannuronic acid (M blocks) and alternate residues of D-guluronic acid and D-mannuronic acid (GM blocks) [20]. Fucans are also one of the major constituents of brown seaweed cell walls, and are ramified sulfated polysaccharides constituted by a central backbone of fucose sulfated in positions C2 and/or C4 and ramifications at each two or three fucose residues [21]. 

Red seaweed polysaccharides are primarily classified as agarans and carrageenans based on their stereochemistry, specifically galactans with 4-linked α-galactose residues of the L-series are termed agarans, and those of the D-series are termed carrageenans [22]. Carrageenans are sulfated D-galactans composed of repeating disaccharide units with alternating 3-linked β-D-galactopyranose (G-units) and 4-linked α-galactopyranose (D-units) or 3,6-anhydro-α-galactopyranose (AnGal-units), which possess broad-spectrum antiviral activities [18]. In conclusion, the seaweed polysaccharides are the most abundant polysaccharides in marine plants, and usually possess the special characteristics of high sulfation and carboxylation.

### 2.3. The Main Structure Features of Marine Microbial Polysaccharides

Marine microorganisms, including bacteria, fungi, and microalgae, are of considerable importance as promising new sources of a huge number of biologically active products [23,24,25,26]. Some of these marine species live in high-pressure, high-salt, low-temperature, and oligotrophic environments, which provide the opportunity for them to produce unique active substances that differ from the terrestrial ones [27]. 

Marine microbial polysaccharides, especially the extracellular polysaccharides, have structural diversity, complexity, and particularity. Most of these polysaccharides are heteropolysaccharides which composed by different monosaccharides in a certain percentage, wherein the glucose, galactose and mannose are the most common components in microbial polysaccharides. It was reported that spirulan, a sulfated polysaccharide isolated from *Arthrospira platensis* (formely *Spirulina platensis*) is composed of two types of disaccharide repeating units, [→3)-α-L-Rha(1→2)-α-L-Aco-(1→] where Aco (acofriose) is 3-*O*-methyl-Rha with sulfate groups and *O*-hexuronosyl-rhamnose [28].

In addition, the marine microbial polysaccharides also contain glucuronic acid, galacturonic acid, amino sugars, and pyruvate. Roger *et al.* [29] reported that the exopolysaccharide (EPS) derived from marine bacterium *Alteromonas infernus* is a highly branched acidic heteropolysaccharide with a high molecular weight and low sulfate content (less than 10%). Its nonasaccharide repeating unit is composed of uronic acid (galacturonic and glucuronic acid) and neutral sugars (galactose and glucose), and substituted with one sulfate group [29]. In conclusion, the marine microbial polysaccharides with novel chemical compositions and structure features have been found to possess potential applications in fields such as pharmaceuticals, food additives, and industrial waste treatments.

## 3. The Antiviral Effects and Structure–Activity Relationships of Marine Polysaccharides

### 3.1. The Antiviral Activities of Carrageenan Polysaccharides and Their Oligosaccharide Derivatives

Carrageenans are one of the major constituents of red seaweed cell walls representing 30% to 75% of the algal dry weight [30]. Carrageenan is mainly extracted from certain genera of red seaweeds such as *Chondrus*, *Gigartina*, *Hypnea*, and *Eucheuma*, and is currently the most widely studied red algae polysaccharide [31]. Carrageenan polysaccharides are normally classified according to their structural characteristics, including their sulfation patterns and the presence or absence of 3,6-anhydro bridges in α-linked galactose residues [18].

The main three types of carrageenans are λ-, κ-, and ι-carrageenan, and each show different inhibitory effects on different viruses [32,33,34,35,36,37]. Buck *et al.* demonstrated that carrageenans could inhibit the initial infection process of human papillomavirus (HPV), and the antiviral effect of ι-carrageenan is better than that of λ- and κ-carrageenans [38]. Talarico and co-workers reported that ι-carrageenan could inhibit dengue virus (DENV) replication in mammalian and mosquito cells, and the mode of action of ι-carrageenan in both cell types is strikingly different. [33,37]. Yamada and co-workers reported that *O*-acylated carrageenan polysaccharides with different molecular weights had increased anti-HIV activity by depolymerization and sulfation [39,40]. Thus, the antiviral activities of carrageenan polysaccharides are associated with their molecular weights and the content of sulfates. Despite having good inhibitory effects on virus infection, the high molecular weight (MW) and poor tissue-penetrating ability of carrageenan polysaccharides have limited potential antiviral application in humans.

Carrageenan oligosaccharide prepared from carrageenan by chemical or enzymatic degradation has smaller molecular weight and is easy to contact with viruses, thus its bioavailability and biological activity is significantly increased [41]. Tang *et al.* found that low molecular weight carrageenans and their derivatives showed significant inhibition effects against influenza virus FM1-induced pulmonary edema in mice, and the 3 kDa k-carrageenan with proper acetylation degree and sulfation degree possesses best antiviral activity *in vivo* [42]. Wang and co-workers reported that the low molecular weight carrageenan oligosaccharide KCO and their sulfated derivatives could effectively inhibit influenza A virus (IAV) replication *in vitro* and *in vivo*, and KCO mainly inhibits a replication step in IAV life cycle subsequent to viral internalization but prior to virus release [43,44]. In addition, the structure–activity relationship study of KCO showed that the specific sugar linkage, a certain sugar length and sulfate content might be essential for the anti-IAV activity of k-carrageenan oligosaccharides. The most active k-carrageenan oligosaccharide has a sulfate content of 0.8–1.0 mole/mole of disaccharide and a molecular weight of 1–3 kDa [44].

In conclusion, the antiviral activities of carrageenans are very broad, which can suppress the replication of both enveloped and non-enveloped viruses. The antiviral effects of carrageenans are closely related to the molecular weights and the degree of sulfation of them. Moreover, the inhibitory actions of carrageenans on different viruses are usually different, which are associated with the types of carrageenans, the virus serotypes and the host cell itself [45,46,47,48].

### 3.2. The Antiviral Activities of Heparin and HeparinoidPolysaccharides

Heparin is a member of the glycosaminoglycan (GAG) family of carbohydrates and consists of a variably sulfated repeating disaccharide unit. The most common disaccharide unit is composed of 2-*O*-sulfate-alpha-L iduronic acid and 6-*O*-sulfate-*N*-sulfate-α-D glucosamine linked by 1→4 glycosidic bond. Heparin has been used in clinical practice for nearly 70 years, and is mainly used for anticoagulant and the treatment of thrombotic diseases. In addition to the anticoagulant and antithrombotic activities, heparin also possesses the inhibitory effect on smooth muscle cell proliferation, anti-inflammatory effect, antitumor effect, and the antiviral activities [49,50,51,52]. Some studies indicated that the heparin polysaccharides and the low molecular weight heparin could be used as the natural inhibitors of herpes simplex virus type 1 (HSV-1), and the inhibition of HSV-1 infection by heparin requires a unique sulfation moiety [52,53].

**Figure 1:**
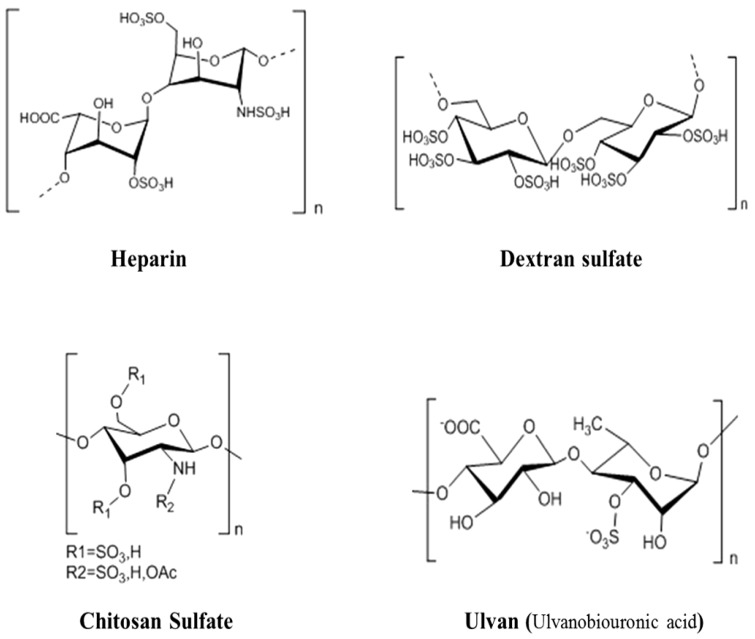
Idealized average structures of the repeat units of heparin and heparinoid polysaccharides [18,38,58,60].

The marine heparinoid polysaccharides are similar to heparin in structure, and possess GAG-like biological properties, which contain alginates, ulvans, and their sulfated derivatives, as well as the dextran sulfate and chitosan sulfate (Figure 1). Recent studies showed that the cell surface heparinoid sulfate proteoglycans are the initial receptors of human herpes virus HSV-1, HSV-2, and bovine herpes virus in their infection processes [54,55,56]. Heparinoid polysaccharides can interact with the positive charge regions of cell surface glycoproteins, leading to the shielding effect on these regions, thus preventing the binding of viruses to the cell surface [54].

Madeleine *et al*. reported that the dextran sulfate could inhibit the fusion of influenza virus with cell membranes, and suppress the replication of influenza virus *in vivo* [57]. Witvrouw *et al*. [58] found that the dextran sulfate mainly inhibits the replication of enveloped viruses, but is inactive against non-enveloped viruses. In addition, the low molecular weight dextran sulfate (DS1000) is virtually inactive against HSV, influenza virus, and some other viruses, and the inhibitory effect of DS1000 against HIV varies considerably depending on the virus strain and the cell type [58]. Moreover, Ivanova *et al.* noted that the ulvan polysaccharides isolated from green algae had good inhibitory effect on influenza A virus, and the inhibition effect is dose-dependent and strain-specific [59]. In addition, another heparinoid polysaccharide—chitosan sulfate also possesses good antiviral activities [60].

In summary, the marine heparinoid polysaccharides have similar pharmacological activities to the natural heparin, and all can inhibit the infection and replication of different kinds of viruses.

### 3.3. The Antiviral Activities of Alginate Polysaccharides and Their Sulfated Derivatives

The principal cell wall polysaccharides in brown seaweeds are alginates and fucans as well as the storage polysaccharide laminarin. Alginate polysaccharides are mainly extracted from brown seaweeds such as kelp, macroalgae, bubble zosteraceae, and marjoram algae, and have been widely used in the food, cosmetic and pharmaceutical industries as a thickener and gelling agent. Alginates are the major constituent of brown seaweeds, which composed of a central backbone of poly-D-glucuronic acid, poly-D-mannuronic acid and alternate residues of D-guluronic acid and D-mannuronic acid [20].

Xin *et al*. reported that a marine polysaccharide drug 911 derived from alginate could significantly inhibit the acute infection of MT4 cells and the chronic infection of H9 cells with HIV-1 [61]. 911 can significantly inhibit the replication of HIV *in vitro* and *in vivo*, and its inhibitory action is attributed to the inhibition of viral reverse transcriptase, the interference with viral adsorption, and the enhancement of immune function [61,62]. Geng *et al.* found that the sulfated polymannuroguluronate (SPMG) could inhibit HIV adsorption mainly through interfering with the interaction of virus gp120 protein with the CD4 molecule on the surface of T cells [63,64,65]. In addition, their studies also indicated that the octasaccharide unit is established to be the minimal active fragment of SPMG inhibiting syncytium formation and lowering the P24 antigen level in HIV-IIIB-infected CEM cells [65]. Moreover, 911 can improve the immune function of host cells, and inhibit the activity of hepatitis B virus (HBV) DNA polymerase, thus 911 can also be used to inhibit the replication of HBV [66].

Fucans are a class of high molecular weight sulfated polysaccharides, which can be classified into three major groups: fucoidans, xylofucoglycuronans and glycuronogalactofucans. They are widely distributed in several species of brown algae and composed of a mainly repeating chain of fucose [60]. The sulfated fucans from the seaweed species *Dictyota mertensii*, *Lobophora variegata*, *Spatoglossum schroederi* and *Fucus vesiculosus* were reported to be able to inhibit the activity of HIV reverse transcriptase (RT) [67]. A fucan polysaccharide from *Cladosiphon okamuranus* composed of glucuronic acid and sulfated fucose units can inhibit the infection of BHK-21 cells with dengue virus type 2 (DENV-2), but shows little effect on the other three serotypes of the virus (DENV-1, DENV-3 and DENV-4) [68]. Sulfation of the fucan is necessary for this activity, and carboxyl-reduction of the glucuronic acids to glucose units abolishes the fucans’ antiviral activities. Moreover, Akamatsu *et al.* reported that one new type of fucose polysaccharides called “MC26,” which is extracted from brown seaweed, has excellent anti-influenza A virus effect *in vitro* and *in vivo* [69].

Laminaran (or laminarin) was first discovered in the laminaria species and appears to be the food reserve of all brown algae. Laminaran is a water-soluble polysaccharide containing 20–25 glucose units composed of (1,3)-β-D-glucan with β(1,6) branching. Laminaran isolated from some brown algae has a good inhibitory effect on virus proliferation. The laminaran polysaccharides prepared from kelp by water extraction were reported to be able to effectively inhibit the adsorption of HIV on lymphocytes and the activity of HIV reverse transcriptase at the concentration of 50 μg/mL, which suggest that laminaran polysaccharides possess good inhibitory effect on HIV replication [70].

In conclusion, brown algae-derived polysaccharides and their derivatives, in particular the alginate polysaccharides and fucans, have good antiviral activities, thus the alginate polysaccharides have the potential to be developed into a new type of antiviral drugs with high efficiency and low toxicity.

### 3.4. The Antiviral Activities of Marine Shellfish Polysaccharides and Their Derivatives

The polysaccharides isolated from marine animals usually have the characteristics of high sulfation, and are also the important resources for the development of antiviral drugs. The marine shellfish polysaccharides are the important animal polysaccharides isolated from marine shellfishes, which commonly including abalone polysaccharides, mussels polysaccharides, and clams polysaccharides. The structure of marine shellfish polysaccharides is very complex, not only because of the wide variety of monosaccharide compositions, but also because of the diversity of linkage types (with or without branches).

Yu *et al.* [71] studied the inhibition actions of scallop skirt glycosaminoglycan (SS-GAG) on the type I-herpes simplex virus (HSV-I) at different concentrations, and they found that SS-GAG has a significant anti-HSV-1 effect *in vitro*. The antiviral activity of SS-GAG gradually increases with the prolonged duration of action [71]. Zhang *et al.* found that marine polysaccharides isolated from *Perna viridis* could significantly inhibit the replication of influenza virus in MDCK cells, and has an additive effect on the anti-IAV actions of ribavirin, which suggest that *Perna viridis* polysaccharides merit further investigation as novel anti-influenza virus agents that can be used alone or in combination with existing antivirals [72].

Moreover, Wu *et al.* reported that the oyster polysaccharides could inhibit the DNA replication of duck hepatitis B virus (DHBV), and reduce the content of DHBV-DNA in duck serum, thus having obvious anti-HBV effects *in vivo* [73]. In addition, Woo *et al.* investigated the inhibition effects of marine shellfish polysaccharides derived from seven kinds of shellfish (*Ruditapes philippinarum*, *Mytilus coruscus*, *Scapharca broughtonii*, *Scapharca subcrenata*, *meretrix lusoria*, *Meretrix petechialis* and *Sinonovacula constricta Lamark*) on the infection of HIV-1 *in vitro*, and the results showed that the seven species of marine shellfish polysaccharides all could inhibit the fusion of virus gp120/gp41 with CD4 protein on the surface of T lymphocytes, of which the *Meretrix petechialis* polysaccharide possesses the most significant anti-HIV activity [74].

In conclusion, a variety of marine active polysaccharides with novel structures and unique functions have been obtained from marine shellfishes, and these polysaccharides are expected to become the new resources for the antiviral drugs development.

### 3.5. The Antiviral Activities of Chitosan Polysaccharides and Their Derivatives

Chitosan, a partially deacetylated polymer of *N*-acetylglucosamine, is produced commercially by deacetylation of chitin [9]. Chitosans were found to effectively reduce the infectivity of feline calicivirus FCV-F9 and bacteriophages MS2 and phiX174. Reduction of MS2 infectivity by chitosan increases as the molecular weight of chitosan increases, with high molecular weight chitosan (over 200 kDa) being able to completely reduce titers of MS2 [75]. Moreover, chitosan was reported to be able to inhibit the systemic propagation of viruses and viroids throughout the plant and enhance the host’s hypersensitive response to infection [76,77,78,79]. In addition, the level of suppression of plant virus infections varies according to the species of plants and the chitosan sources [78]. Furthermore, it was reported that the antiviral activities of the low molecular weight chitosans produced by enzymatic degradation significantly increase with the lowering of their polymerization degree [77,79].

Chemical modification of chitin and chitosan can generate novel compounds that possess good pharmacological properties such as antiviral activities. Sosa *et al.* reported that the *N*-carboxymethylchitosan *N*,*O*-sulfate (NCMCS), a polysaccharide derived from *N*-carboxymethyl chitosan by sulfation modification, could prevent HIV-1 infection by inhibiting viral adsorption to the CD4 receptor and reverse transcription of the viral genome [80]. Moreover, Nishimura *et al.* found that the chitin sulfate had good inhibitory effect on HIV-1 infection, and its inhibition action on HIV-1 depends significantly on the sites of sulfation [81]. In addition, it was reported that the aminoethyl-chitosan, prepared from 50% deacetylated chitosan also shows good inhibitory activity against HIV-1 *in vitro* [60]. 

To improve the water-solubility and biological activity, chitosan also can be converted into chitooligosaccharides (COSs) via either chemical or enzymatic hydrolysis [82,83,84]. Sulfated chitooligosaccharides (SCOS), prepared by a random sulfation modification of chitooligosaccharides, have been reported to possess good anti-HIV activities at low molecular weight (3–5 kDa) [85]. SCOS shows inhibitory actions on viral entry and virus–cell fusion via blocking the interaction between HIV-1 gp120 and CD4^+^ cell surface receptors. Moreover, the administration of chitooligosaccharides on different leaf surfaces can decrease the number of local necroses, which is virus-induced infection [86].

In summary, chitosan polysaccharides and low molecular weight oligosaccharides, as well as their sulfated derivatives, possess good antiviral activities, and their antiviral actions are related to the molecular weights and their sites of sulfation.

### 3.6. The Antiviral Activities of Polymannuronic Acid Polysaccharides and Their Derivatives

**Figure 2:**
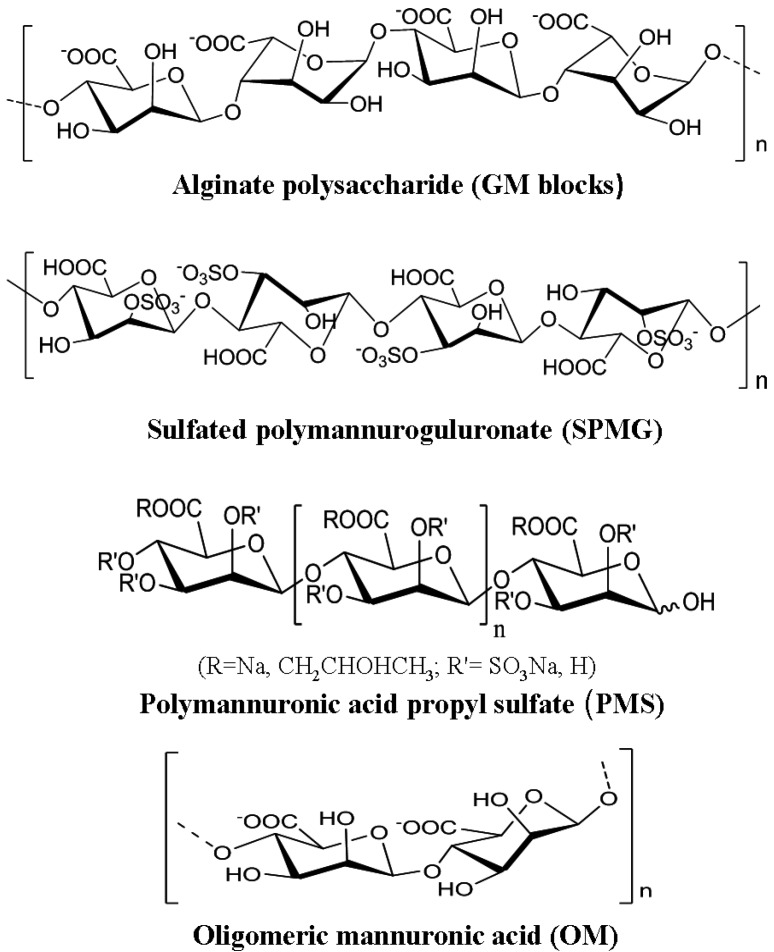
Chemical structures of alginate polysaccharides and their derivatives [20,64,87].

Alginates are linear acidic polysaccharides constituted by a central backbone of poly-D-glucuronic acid (G blocks), poly-D-mannuronic acid (M blocks) and alternate residues of D-guluronic acid and D-mannuronic acid (GM blocks) [20]. The alginate polysaccharide dominated by heterogeneous fragments (MG) has been developed into the novel anti-HIV marine drug 911, which has entered Phase II clinical study [62,64]. The alginate polysaccharide composed of homogeneous fragments (M blocks) has been developed into the anti-cardiovascular marine drug polymannuronic acid propyl sulfate (PMS) [87]. Polymannuronic acid polysaccharides are alginic acid sugar fragments (M blocks) prepared from alginate polysaccharides by enzymatic or acidic degradation and PH fractionation method. The chemical structures of alginate polysaccharides and their derivatives are illustrated in Figure 2.

Polymannuronic acid polysaccharides and their derivatives have heparin-like structures, and also possess good antiviral activities. It was reported that one derivative of polymannuronic acid polysaccharides—polymeric mannuronic acids sulfate that was prepared by the sulfation of the polymannuronic acid fragment—possesses good antiviral effects *in vivo* [88]. Guan and co-workers found that polymeric mannuronic acids sulfate could significantly improve the humoral and cellular immune function, and effectively reduce the surface antigen levels and the DNA levels of hepatitis B virus in duck blood, suggesting that this polysaccharide has good anti-duck HBV effect [88].

Furthermore, it was reported that one oligosaccharide derivative of polymannuronic acid polysaccharide—oligomeric mannuronic acid (OM) with low molecular weight (less than 5 kDa) has good inhibitory effects on the infection of influenza A (H1N1) virus *in vitro* and *in vivo* [89]. Guan and co-workers reported that another derivative of polymannuronic acid—polymannuronic acid propyl sulfate (PMS) could be prepared by the sulfation and propyl modification of polymannuronic acid polysaccharide, which has the heparin-like structural characteristics [87]. Moreover, they also found that PMS has good inhibition effect on the neuraminidase activity of influenza A virus (IAV), and effectively inhibits IAV infection in MDCK cells. Additionally, PMS can effectively alleviate the pneumonia symptom caused by IAV infection, and significantly reduce the mortality rate of mice [90]. 

In conclusion, polymannuronic acid polysaccharides and their oligosaccharide derivatives possess good antiviral activities, which merit further investigation as novel antiviral agents to prevent the infection of HBV and IAV. In order to summarize the data available in the literature, the marine polysaccharides with good antiviral effects described in this paper were all shown in Table 1.

**Table 1:** Antiviral activities of selected marine polysaccharides.

Marine organisms	Specific polysaccharides	Antiviral effects	References
Crustacean	Chitosan	Anti-enteric virus, plant viruses, HIV	[75,76,77,78,79,80,81,85,86]
Red algae	λ-carrageenan	Anti-DENV, HSV, HPV, HIV, HAV	[33,35,38,39,40,46,47]
	κ-carrageenan	Anti-enterovirus, HSV, HIV, IAV	[32,35,39,40,42,43,44]
	ι-carrageenan	Anti-DENV, HRV, IAV, HPV, HAV	[33,34,36,37,38,47,48]
Brown algae	Alginate	Anti-HIV, HBV, IAV	[61,62,63,64,65,66,88,89,90]
	Fucan	Anti-HIV, DENV, IAV	[67,68,69]
	Laminarin	Anti-HIV	[70]
Green algae	Ulvan	Anti-IAV	[59]
Shellfish	Shellfish polysaccharide	Anti-HSV, IAV, HBV, HIV	[71,72,73,74]
Microalgae	Sulfated polysaccharide	Anti-HSV, IAV	[91,92]

## 4. The Mechanisms of Antiviral Actions of Marine Polysaccharides

Marine polysaccharides can either inhibit the replication of virus through interfering viral life cycle or improve the host antiviral immune responses to accelerate the process of viral clearance. The life cycle of viruses differs greatly between species but there are six basic stages in the life cycle of viruses: viral adsorption, viral penetration, uncoating of capsids, biosynthesis, viral assembly and viral release. Marine polysaccharides can inhibit viral life cycle at different stages or directly inactivate virions before virus infection. Specific antiviral mechanism of marine polysaccharides is commonly related to specific structure features of the polysaccharides and specific viral serotypes [45].

### 4.1. Direct Virucidal Action

Carrageenan, a sulfated polysaccharide, might inhibit virus infection via direct actions on the virus surface by its negative charge. Several studies showed that carrageenan has a direct virucidal action on some enveloped viruses, which makes the viruses lose the ability to infect cells, thus effectively reducing the virus multiplication. Carlucci *et al.* found that λ-type carrageenan could firmly bind to the herpes simplex virus (HSV), leading to the inactivation of theHSV virion, thus inhibiting the replication of HSV [93]. Their studies further suggest that carrageenan changes the structure of the glycoproteins gB and gC of HSV [46,93].

Moreover, Harden *et al.* [94] reported that carrageenan polysaccharides derived from red algae could directly inactivate HSV-2 at low concentrations. The virucidal activities increase with increased molecular weight of carrageenan polysaccharide up to 100 kDa, after which the virucidal activities level off [94]. The direct virucidal actions of carrageenan may be due to the formation of a stable virion–carrageenan complex where binding is not reversible and hence the sites on the viral envelope required for virus attachment to host cells are occupied by the sulfated polysaccharide, which renders the virus unable to complete the subsequent infection process [45].

In addition, chitosan and chitosan oligosaccharides were reported to be able to effectively reduce the infectivity of two human enteric viral surrogates: feline calicivirus F-9 (FCV-F9), and bacteriophage MS2 (MS2) by incubating the two viruses with chitosan or chitosan oligosaccharides for 3 h at 37 °C before infection [75,95]. The inhibition actions might be due to the direct inactivation of these two enteric viral surrogates by chitosan. Furthermore, reduction of MS2 infectivity by chitosan increases as the molecular weight of chitosan increases, while the inhibitory effects of chitosan on FCV-F9 is not MW-dependent [95]. 

In conclusion, chitosan and carrageenan polysaccharides all have direct virucidal actions on some viruses to block the virus infection. This virucidal activity is important for marine polysaccharides since it is associated with the augmentation of antiviral activities *in vivo* [35].

### 4.2. Inhibition of Viral Adsorption

The first step of virus invasion into host cells is binding to host cell surface via electrostatic interactions, and then transforming unstable reversible binding into stable irreversible adsorption in order to achieve subsequent invasion process. Some marine polysaccharides can interfere with viral adsorption process either by interacting with virions or by interacting with virus receptors on the host cell surface.

Several studies have shown that carrageenan can mask the positive charge of host cell surfaces by the negative charge of its sulfate groups, so as to interfere with the adsorption process of viruses. Mazumder *et al.* obtained a high molecular weight sulfated galactan from red algae, and showed its antiviral activities against herpes simplex virus 1 and 2 in bioassays, which is likely due to an inhibition of the initial viral attachment to the host cells [96]. Carlucci *et al*. noted that λ-carrageenan and partially cyclized μ/ι-carrageenan from *Gigartina skottsbergii* have potent antiviral effects against different strains of HSV types 1 and 2 during the virus adsorption stage [97]. They subsequently confirmed the firm binding of carrageenan to virus receptors on the host cell surface. Their studies demonstrate that λ-carrageenan interferes with the adsorption process of the virus to the host cell surfaces [97,98].

Fucoidan isolated from brown algae has good antiviral activities against viruses such as HIV [99], HSV and human cytomegalovirus [100,101,102,103]. The antiviral actions of fucoidan seem to stem from inhibiting the binding of the virus particles to the host cell [103]. In addition, sulfated polymannuroguluronate (SPMG), a new form of marine polysaccharide extracted from brown algae, could inhibit HIV-1 adsorption and entry by sharing common binding sites on gp120 with sCD4 or masking the docking sites of gp120 for sCD4 on the surface of T lymphocytes [64].

Furthermore, one of the chitosan derivatives, *N*-carboxymethylchitosan *N*,*O*-sulfate (NCMCS), could inhibit the replication of HIV-1 by blocking the interactions of viral coat glycoprotein receptors with target proteins on lymphocytes [80]. In addition, Kanekiyo *et al.* [91] reported that the acidic polysaccharide nostoflan isolated from the edible blue-green alga *Nostoc flagelliforme* have a good inhibitory effect on HSV-1. Their results indicate that the inhibition of virus binding to—but not penetration *into*—host cells is responsible for the antiherpetic effect induced by nostoflan [91]. In summary, marine polysaccharides from different sources can block virus infection by interfering with the virus adsorption process. 

### 4.3. Inhibition of Virus Internalization and Uncoating

The internalization process of most animal viruses often involves the endocytic uptake, vesicular transport through the cytoplasm, and delivery to endosomes and other intracellular organelles [104]. The uncoating step of the viral life cycle usually occurs after viral internalization into host cells, while the uncoating of some phages occurs in the same time of internalization. Certain marine polysaccharides, especially sulfated marine polysaccharides, can interfere with virus internalization and uncoating by blocking the allosteric process of virus particles.

Buck *et al.* found that carrageenan could directly bind to the HPV capsid, so as to inhibit not only the viral adsorption process but also the subsequent entry and uncoating process of the virus [38]. They also found that the inhibition actions of carrageenan against HPV might be related to a mechanism that is independent of the heparan sulfate after viral adsorption [38]. Moreover, Talarico and co-workers reported that λ- and ι-carrageenans could interfere with both DENV-2 adsorption and internalization into host cells and are only effective if added together with the virus or shortly after infection [33]. The mechanism of this inhibition action may be due to that although DENV virus can enter into host cell in the presence of carrageenans, their subsequent uncoating and releasing from endosomes may be interfered by the carrageenans. The inhibition action of ι-carrageenan on the uncoating process of dengue virus may be attributed to the direct interaction of carrageenans with the virus membrane glycoprotein E (gE) [33,48,105]. 

Moreover, Grassauer *et al.* reported that ι-carrageenan could inhibit rhinovirus (HRV) multiplication by interfering with the very early stages of virus replication, and this inhibition is probably due to the interference of the allosteric process of virus particles during HRV internalization [34]. Furthermore, Kim *et al.* reported that the sulfated polysaccharide, p-KG03, purified from the marine microalga, *Gyrodinium impudium*, exhibit good inhibitory effect on influenza A virus infection [92]. The mechanism studies showed that the inhibition of virus replication is maximized when p-KG03 is added during or within 6 h after viral infection, suggesting that mainly the viral adsorption and internalization steps are targeted by this compound. Thus the marine polysaccharide p-KG03 cannot only inhibit the binding of influenza virus to host cells, but also prevents the cellular internalization of the virus and early viral replication [92].

In conclusion, some marine polysaccharides can interact with virus membrane glycoproteins to interfere with virus internalization and the subsequent uncoating and releasing from endosomes in the cytoplasm in order to inhibit virus multiplication *in vitro*. 

### 4.4. Inhibition of Virus Transcription and Replication

Marine polysaccharides, especially the low molecular weight marine oligosaccharides, cannot only block the virus invasion process, but also inhibit the virus transcription and replication processes after internalization into host cells. The inhibition of virus transcription and replication may be related to the direct interference of viral replication enzymes or on other intracellular targets by marine polysaccharides.

Gonzalez *et al.* used [^35^S] methionine-labeled virions to analyze the internalization of HSV-1 into host cells, and found that carrageenan polysaccharides have no inhibitory effect on virus adsorption or virus entry, but the subsequent protein synthesis of virus is declined [106]. However, the expression of viral late proteins could not be affected by the carrageean polysaccharides if the compounds are added to host cells one hour after virus infection, which suggest that this sulfated polysaccharide inhibits a step in virus replication subsequent to viral internalization but prior to the onset of late viral protein synthesis [106]. In addition, Talarico *et al.* found that ι-carrageenan could inhibit the replication of DENV in mosquito cells. Its mechanism of action is not to suppress the virus adsorption process but to affect the potential targets within the host cells [37].

Moreover, carrageenan oligosaccharides with low molecular weights can also inhibit virus transcription and replication process after entering into host cells. Wang *et al.* found that κ-carrageenan oligosaccharide with low molecular weight could effectively inhibit influenza A H1N1 virus replication *in vitro* and *in vivo* [43,44]. They also noted that carrageenan oligosaccharide CO-1 does not bind to the cell surface of MDCK cells and does not interfere with the IAV adsorption and internalization process directly [43]. Different to the actions of carrageenan polysaccharides, the oligosaccharide CO-1 could enter into host cells and inhibit IAV mRNA transcription and protein translation after its internalization. In a word, carrageenan oligosaccharide CO-1 might inhibit an early replication step of virus life cycle subsequent to IAV internalization [43]. 

In addition, some marine polysaccharides derived from brown algae can also inhibit virus replication in host cells. Queiroz *et al.* found that fucans isolated from *F. vesiculosus* have a pronounced inhibitory effect *in vitro* on the HIV reverse transcriptase at a concentration of 0.5–1.0 mg/mL, and the inhibitory effect of fucans is eliminated by their desulfation [67]. Queiroz and co-workers also indicated that the antiviral activity of fucans is not only dependent on the ionic changes but also on the sugar rings that act to spatially orientate the charges in a configuration that recognizes the enzyme, thus determining the specificity of the binding [67]. In addition, the alginate derivative 911 can also inhibit effectively the activity of HIV-1 reverse transcriptase, in addition to its inhibition of virus adsorption [61]. 

In conclusion, marine polysaccharides, especially the seaweed polysaccharides, can interfere with virus replication enzymes [61,67] or other potential targets within the host cells [37], so as to inhibit the virus transcription and replication processes.

### 4.5. Improvement of Host Antiviral Immune Responses

The virus infection can induce the antiviral immune responses of host cells, of which the type I interferon system (IFN-α/β system) is the cell’s first line of defense against viral infections. Moreover, the host NK cells and macrophages also play important roles in these processes. Therefore, if marine polysaccharides can activate NK cells and macrophages, or stimulate the generation of antiviral immune factors, they will be able to indirectly inhibit virus replication and accelerate the process of viral clearance.

Turner *et al.* found that λ-carrageenan polysaccharides could significantly induce the synthesis of type I interferon in mice after being treated with carrageenan for eight hours [107]. Zhou *et al.* reported that the λ-carrageenan polysaccharides prepared by microwave degradation could significantly improve the activity of NK cells, and enhance the proliferation rate of lymphocyte [108]. Moreover, Yuan *et al.* noted that carrageenan oligosaccharides could also enhance the activities of macrophages and NK cells and increase the expression of IL-2 and TNF-α [109]. Therefore, the antiviral effects of carrageenans *in vivo* might also be related to their stimulating effects on host immune system.

Moreover, certain polysaccharides derived from brown algae and green algae can also improve the antiviral immune responses of host. The sulfated polymannuroguluronate (SPMG) derived from brown algae was reported to be able to significantly improve the humoral and cellular immune functions to indirectly inhibit the replication of HIV and HBV [62,66]. Xin *et al.* noted that the alginate polysaccharide drug 911 could enhance the proliferative response of T and B lymphocytes in mice, and strengthen the capacity of spleen cells to produce IL-2 and hemolysin antibodies [62]. In addition, water-soluble sulfated polysaccharides (SP) extracted from *Enteromorpha prolifera* were reported to be able to increase the IFN-γ and IL-2 secretions, which suggested that SP could activate T cells by upregulating Th-1 response [110]. In a word, several marine polysaccharides can inhibit virus replication and accelerate the process of viral clearance indirectly through activating the immune system.

## 5. Conclusions

During the last decade, numerous bioactive polysaccharides with interesting functional properties have been discovered from marine organisms [111]. Marine polysaccharides, especially the sulfated polysaccharides derived from marine algae, often possess good inhibitory effects on a variety of viruses [18,112]. This review mainly focuses on the antiviral activities and mechanisms of marine polysaccharides, which is expected to attract more interest for future explorations. The antiviral activities of most marine polysaccharides are usually related to the specific sugar structure, molecular weights and their degree of sulfation. Marine polysaccharides can inhibit the replication of viruses through interfering with a few steps in virus life cycle or improving the host antiviral immune responses to accelerate the process of viral clearance. Despite having good antiviral effects, marine polysaccharides are structurally diverse and heterogeneous, which makes studies of their structures challenging, and may also have hindered their development as therapeutic agents to date [18].

In conclusion, marine polysaccharides, especially the polysaccharides derived from seaweed, have many advantages, such as relatively low production costs, broad spectrum of antiviral properties, low cytotoxicity, and wide acceptability, which suggest marine polysaccharides merit further investigation as promising antivirals that can be used alone or in combination with existing drugs [113]. Until now, most of the studies on antiviral effects of marine polysaccharides have been observed *in vitro* or in mouse model systems. Therefore, further studies are needed in order to investigate their antiviral activities in human subjects [60]. Moreover, the structure–activity relationships and the underlying molecular mechanisms of antiviral actions of marine polysaccharides need to be understood precisely and elucidated clearly by intensive studies in the future [111].
